# Comparison of laser and intense pulsed light sintering (IPL) for inkjet-printed copper nanoparticle layers

**DOI:** 10.1038/srep08832

**Published:** 2015-03-06

**Authors:** Juha Niittynen, Enrico Sowade, Hyunkyoo Kang, Reinhard R. Baumann, Matti Mäntysalo

**Affiliations:** 1 Department of Electronics and Communications Engineering, Tampere University of Technology, Tampere, Finland; 2 Digital Printing and Imaging Technology, Technische Universität Chemnitz, Chemnitz, Germany; 3 Department Printed Functionalities, Fraunhofer Research Institute for Electronic Nano Systems (ENAS), Chemnitz, Germany

## Abstract

In this contribution we discuss the sintering of an inkjet-printed copper nanoparticle ink based on electrical performance and microstructure analysis. Laser and intense pulsed light (IPL) sintering are employed in order to compare the different techniques and their feasibility for electronics manufacturing. A conductivity of more than 20% of that of bulk copper material has been obtained with both sintering methods. Laser and IPL sintering techniques are considered to be complementary techniques and are highly suitable in different application fields.

The sintering of metallic nanoparticle inks in the field of printed electronics has been studied in recent years extensively using different materials and sintering techniques^1^,^2^,^3^,^4^,^5^,^6^,^7^,^8^,^9^,^10^. The focus has previously been set mainly on silver nanoparticles as they have been, and still remain, the most commonly used conductive material in printed electronics. However, the costs for silver are very high and thus a lot of effort has been done to move the focus towards alternative materials based on cheaper bulk metals such as copper, aluminum and nickel. Today, ink formulations based on copper nanoparticles are already commercially available and receive increasing interest^11^,^12^,^13^,^14^. Aluminum and nickel nanoparticle inks are still under development. Therefore, in this contribution a copper nanoparticle ink for inkjet printing was chosen. The challenges when using these alternative metals are the development of nanoparticles with a small size distribution stably dispersed in a solvent and the post-processing, in particular the sintering, of these particles.

The processing of copper nanoparticles is much more demanding than that of silver or gold because of the high rate of oxidation and the higher melting point of copper compared to silver and gold. Unlike silver oxides, copper oxides are non-conductive and therefore the oxidation of copper during sintering processes prevents the formation of electrically functional structures. This puts significant requirements for the processing speed of the sintering process or for the ambient atmosphere. Conventional thermal sintering in oven or on hotplate as well as some of the alternative sintering techniques, such as plasma sintering, are too slow and the copper nanoparticles will oxidize during the sintering process in ambient condition. Furthermore, high temperatures are required for the thermal sintering process which is not compatible with cost-effective polymer foils frequently used in printed electronics such as PET and PEN. In contrast, laser and IPL sintering techniques offer rapid and high energy sintering processes so that the oxidation can be neglected and temperature sensitive polymer foils can be employed as substrates.

The sintering of copper nanoparticles using laser or IPL has been studied in literature before but a comprehensive comparison of both techniques has not yet been done. To analyze similarities and differences and therefore also advantages and limitations of the different sintering methods, a comparative study utilizing the same materials and diagnostic methods is required.

Both laser and IPL sintering techniques rely on the absorption of emitted photonic energy by the inkjet-printed nanoparticle layers. But the operating methods of laser and IPL sintering differ significantly. Laser sintering is based on a small spot size (usually in mm^2^ scale) due to coherent light emission with high energy density on a fixed wavelength. Processing of larger areas is achieved by scanning the small laser spot over the area to be treated. IPL sintering on the other hand relies on a scalable xenon flash lamp emitting light over a wide wavelength spectrum. IPL sintering affects much larger areas at once in comparison to laser sintering (usually in cm^2^ scale), and is therefore better suited for larger patterns e.g., for roll-to-roll printing and large area electronics. On the other hand, laser sintering will not affect other parts than these ones scanned and is thus a digital patterning technique itself. Sintering can be done at different positions with high accuracy and varying intensities resulting e.g. in different conductivities. In this so-called “grayscaling” mode the power of the laser is adjusted depending on the pattern to be treated. Combined with the digitally controlled and highly customizable inkjet-printing process, it enables a flexible fabrication of devices with localized sintering requirements, e.g. patterns with large and extremely fine feature sizes in the same pattern. In this way, laser sintering is much more flexible in comparison to IPL sintering. However, due to the scanning approach of the laser it is rather slow – especially for larger areas. As a reference, processing an A4-sized sheet with the laser sintering equipment used in this study takes about 10 to 15 minutes compared to maximum a few seconds with IPL sintering. Therefore, both sintering techniques are considered to be complementary methods for each other. They are compared in this research in detail by the usage of the same inkjet deposition method, the same ink formulation and the same substrate. This allows setting the focus on the individual sintering techniques and its adjustable parameters.

## Results and Discussion

### Laser sintering

**Figure 1:**
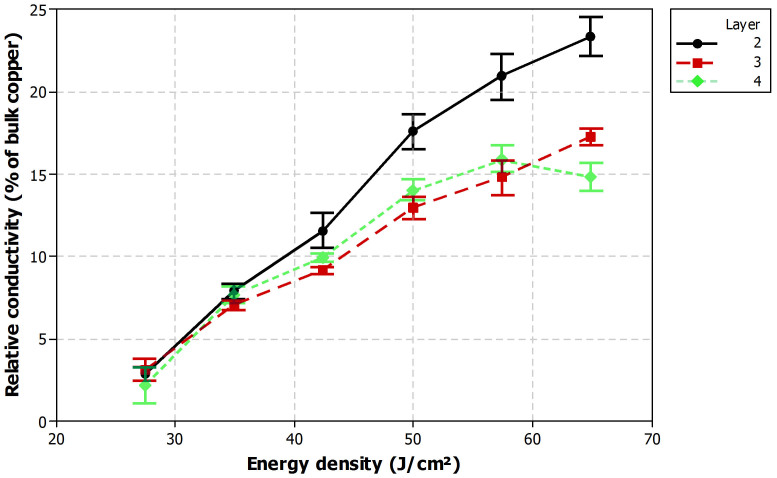
Relative conductivity as a function of energy density for two, three and four layers in laser sintering with a scanning speed of 100 mm/s.

After printing, the dried layers were treated with the laser with rastered processing. The influence of the energy density of the laser and its scanning speed on the conductivity of the printed layers was investigated. Figure 1 presents the obtained relative conductivity as a function of energy density for all tested layer counts using laser sintering and a scanning speed of 100 mm/s. Each point presents an average of 10 measurement results and 95% confidence interval is shown in the plot. It can be clearly seen, that the conductivity increases as energy density is increased. This is expected as higher energy density results in higher sintering temperature and thus improved removal of organic stabilizers and better fusion of copper nanoparticles.

The difference between the number of layers is also significant: two layer seem to enable substantial higher relative conductivity compared to three and four layers. However, the difference between three and four layer samples is insignificant. Even though two layer samples have lower relative conductivity, the three and four layer samples have lower overall resistance due to a larger conductor line cross-section. One layer samples were also tested but no conductivity was obtained because intensive cracking appeared in the thin copper layer during laser sintering.

**Figure 2:**
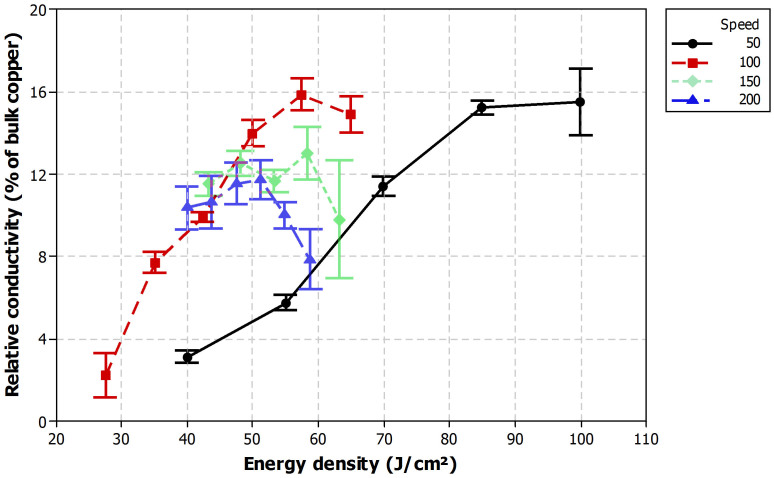
Relative conductivity as a function of energy density for different laser scanning speeds with four layer patterns.

Figure 2 shows the relative conductivity exemplarily for four layers as a function of energy density for laser scanning speeds of 50, 100, 150, and 200 mm/s. Each point represents an average of 10 measurement results and 95% confidence interval is included in the plot. It was found, that the laser scanning speed is one of the important factors in the sintering process. The highest relative conductivity of about 16% of bulk copper was obtained with 57.4 J/cm^2^ for a scanning speed of 100 mm/s. However, increasing the energy density beyond a certain threshold results in lower relative conductivity indicating more layer defects such as cracks due to the high energy. The same trend can be also observed for 100, 150 and 200 mm/s scanning speeds. In the case of 50 mm/s, the relative conductivity increases continuously even for higher energy densities. The reason is that the copper is exposed for a longer time to the laser radiation less abruptly in comparison to higher speeds and therefore less stress cracks will occur. From Figure 2 it is obvious, that the energy density alone cannot be used to determine or predict the electrical conductivity as other parameters also affect. For example, Figure 2 shows that energy density just below 60 J/cm^2^ results to about 16%, 13%, and 8% relative conductivity with scanning speeds of 100, 150, and 200 mm/s, respectively.

### Microstructure

**Figure 3:**
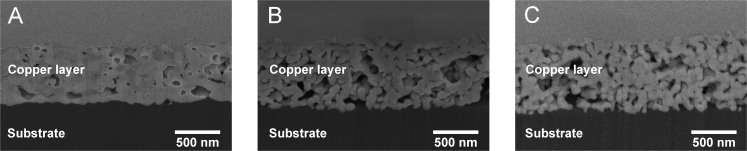
Cross section of four printed copper layers laser-sintered with (A) 84.9 J/cm^2^ and 50 mm/s scanning speed, (B) 64.9 J/cm^2^ and 100 mm/s scanning speed and (C) 51.1 J/cm^2^ and 200 mm/s scanning speed. The test sample details are listed in Table 1.

In addition to electrical performance the microstructure of the sintered layers was investigated with SEM utilizing FIB technology for cutting an observable cross-section. Figure 3 shows the SEM images of laser-sintered samples with four layers. Corresponding image details are listed in Table 1. For a high energy density of 84.9 J/cm^2^ the copper nanoparticles are well agglomerated and form larger grain sizes than that of less energy densities. Obviously, there is very little difference between Figure 3 B) and C), but Figure 3 A) has a significantly more uniformal microstructure with less pores. However, electrical conductivity of samples in Figures 3 A) and B) are similar, whereas the sample in Figure 3 C) has significantly lower conductivity. More uniformal microstructure is most likely due to increased energy density although the microstructure does not seem to correlate in this case directly with the achievable conductivity. The shown cross-section of microstructure is a very small detail of a macroscopic layer. Macroscopic phenomena such as crack formation are not considered in the images but they also influence the conductivity remarkably.

### IPL sintering

The IPL sintering setup offers a wide range of processing parameters which affect the sintering performance. The energy density is one of the most important factors mainly determined by the bank voltage and the pulse duration. The energy density used in this publication is considered as the surface exposure underneath the flashlamp. The value was not measured but is an estimation given by the software PulseForge 1.1 for the used pulse settings and the physical parameters of the flashlamp bulbs. In reality, the exposure energy of the IPL tool on the test samples is smaller than the given energy density, e.g. because the distance between the bulbs and the samples being processed is not considered in the software. Next to the energy density, also the number of pulses and the delay between the pulses (pulse frequency) have an influence on the sintering properties as we will show in following.

### Single vs multi pulse

The PulseForge IPL sintering unit can be used either with single pulse setup, where the printed sample is exposed to only a single flash, or multi pulse setup, where at least two consecutive flashes are applied. Therefore, within this contribution the term pulse refers to flash. Each pulse results in an individual flash. When using multi pulses, the repetition frequency can be adjusted. Using the multi pulse mode, the processed sample can have (i) enough time to cool down between the pulses if the frequency is very low or (ii) will heat up more in a stepwise manner at high frequencies (see S1 and S2 in Supplementary Information). However, applying high frequencies (multi pulses with only a short time delay between each other) will result in decreasing exposure energy densities from pulse to pulse due to capacitor charging limitations, but within the sample the temperature can rise up with each single flash. For low frequencies the sample will heat and cool down with each flash and thus the temperature within the sample is similar for each flash. (For further information see S1 and S2 in Supplementary Information)

**Figure 4:**
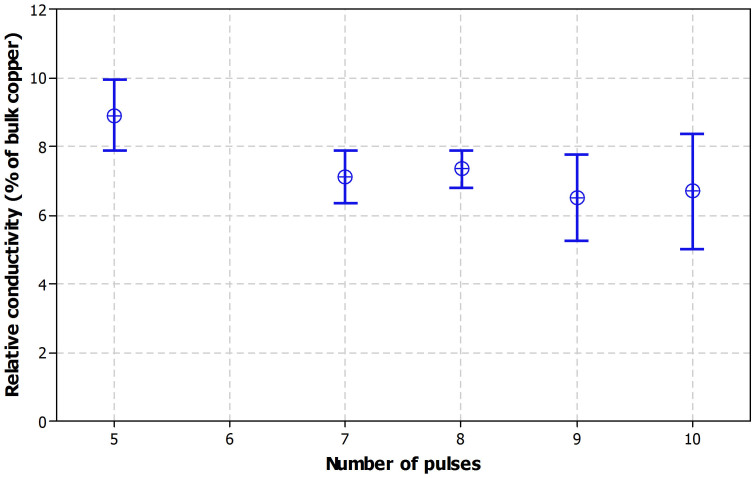
Relative conductivity as a function of number of pulses; each pulse has a duration of 1 ms and the repetition frequency is 60 Hz at 320 V.

Several numbers of pulses were tested in this study and the achieved relative conductivity was compared. Figure 4 presents the relative conductivity as a function of number of pulses for two layer samples with a lamp driver voltage of 320 V and a pulse duration of 1 ms per pulse at 60 Hz. A similar behavior was observed for all the other numbers of layers as well. Slightly higher conductivity was achieved with the five pulse setup but there seems to be no significant difference between 7, 8, 9 and 10 pulse setups. Each result is an average of six measurements and the plot includes 95% confidence intervals.

Another comparison is made between different pulse frequencies as it affects the cooling time between pulses. Frequency variations were done from 10 to 60 Hz with two and five consecutive pulses in order to see if changing the pulse frequency affects the conductivity.

**Figure 5:**
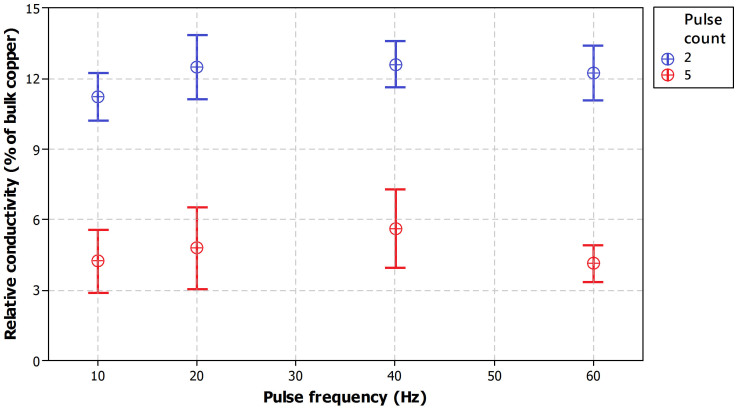
Comparison of relative conductivity as a function of pulse frequency for four layer samples with two and five pulse repetition setups at 320 V and a theoretical energy density of approximately 8.94 J/cm^2^.

Figure 5 shows the relative conductivities as a function of pulse frequency for both two pulse and five pulse setups. Each result represents an average of 12 measurements and 95% confidence intervals are included in the plot. The pulse duration was varied to compensate for pulse count so that in theory the overall pulse time in both cases remained constant at 5 ms. Also the lamp driver voltage was kept the same (at 320 V) for all test samples. Thus, the overall theoretical energy density for both cases is the same. However, for 1 ms pulse duration and five pulses the final real energy density is slightly higher than two pulses both with 2.5 ms. The reason for the slight difference is the slope of the voltage over time (see S3 and S4 in Supplementary Information). The graph in Figure 5 only includes four layer samples but similar behavior was observed with one to three layers as well. It can be seen, that the number of pulses has a significant influence on the relative conductivity whereas the frequency has only a minor influence on the relative conductivity. Higher relative conductivities were obtained for all frequencies and all number of layers with two pulses. The samples with five pulses show nearly three times lower relative conductivity. We ascribe the difference to layer defects that seem to increase with a higher number of pulses. The higher number of pulses results in stress promoting the crack formation in the layer. As a consequence, lower relative conductivity was obtained for five pulses.

### Bank voltage effect

**Figure 6:**
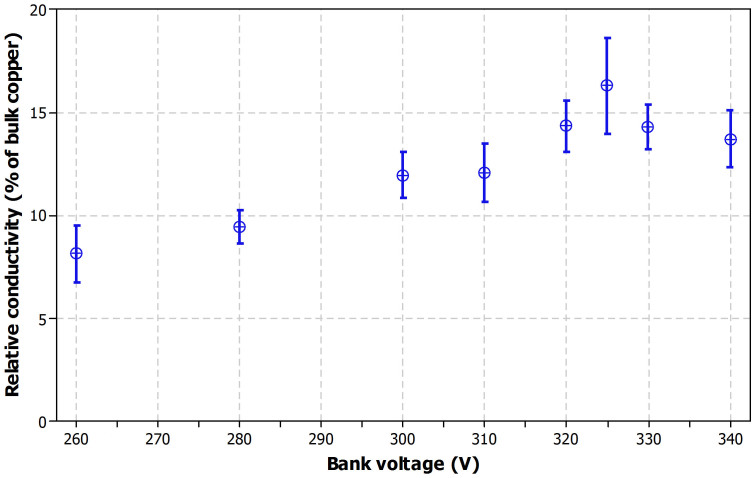
Relative conductivity as a function of bank voltage for one 5 ms pulse and two layers printed.

The energy density of the PulseForge IPL sintering unit is mainly influenced by the bank voltage. Figure 6 presents the relative conductivity as a function of bank voltage for single pulse setups for two layer samples, all with 5 ms pulse duration. Each result represent an average of six measurements and 95% confidence intervals are included in the plot. The lowest tested voltage levels (below 300 V) result in relatively poor conductivity and the conductivity increases as bank voltage is increased until a peak value of 16.3% relative conductivity is reached with 325 V. Increasing the voltage beyond this threshold voltage of 325 V reduces the conductivity due to intensive macroscopic crack formation.

### Energy density

**Figure 7:**
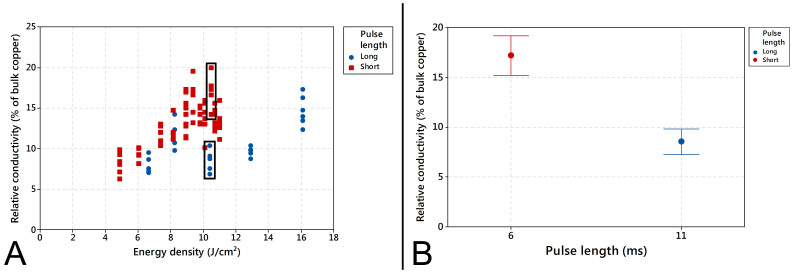
(A) Scatterplot of relative conductivity versus energy density for two layer patterns with single pulse setup for short (5 ms and 6 ms) and long (8 ms and 11 ms) pulse lengths (thus bank voltage and pulse duration vary and same energy density values are obtained with different combinations of bank voltage and pulse duration values). (B) Interval plot of single set of short (6 ms) and long pulse length (11 ms) samples with comparable energy density.

The energy density is the result of all parameters introduced before. It is the surface exposure underneath the flashlamp. Figure 7 presents the conductivity data for different energy densities. Figure 7 A) shows a scatterplot of relative conductivity versus energy density of two layer samples sintered with single pulse IPL setups for both short (5 ms and 6 ms) and long (8 ms and 11 ms) pulse length setups. Each point represents a single measurement. However, due to measurement resolution several measurements are overlaid in the plot. From Figure 7 A) it can be seen, that even though the energy density has an effect on the obtained relative conductivity, it is far from being the only explaining factor. This is a similar observation as done earlier for the laser sintering (see Figure 2). The conductivity can be affected not only by the energy density, but also strongly depends on the bank voltage and pulse duration (as well as the number of pulses and frequency). This graph indicates, that displaying the energy density of IPL sintering given by the IPL system is not sufficient process information. E.g., energy density of about 10.6 J/cm^2^ can result in relative conductivities from about 6% up to about 20% of bulk copper depending on other parameters (see marked area in Figure 7 A), e.g. bank voltage and pulse length. Overall, a trend can be observed that higher energy density results in higher relative conductivity and that short pulses also result in higher relative conductivities compared to long pulses. Figure 7 B) presents an interval plot of the measurement data of a single parameter set for both short (6 ms) and long (11 ms) pulse lengths. The parameter sets have been chosen for having comparable energy densities (10.5 J/cm^2^ for short and 10.4 J/cm^2^ for long pulse length) and yet there is significant difference in the relative conductivity. From Figure 7 B), it can be seen that same energy density results into two distinctively different conductivity results. Bank voltages and pulse lengths used in test cases presented in Figure 7 B) are 325 V and 260 V and 6 ms and 11 ms for short and long pulse length respectively.

### Number of printed layers

**Figure 8:**
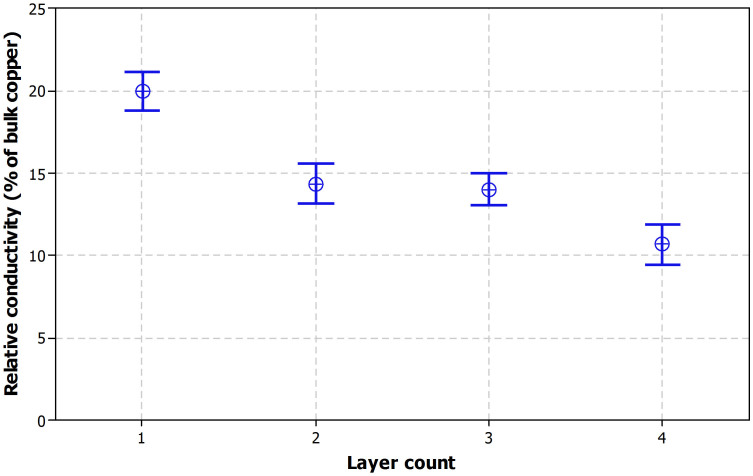
Relative conductivity as a function of printed layer count at 320 V and a pulse duration of 5 ms (single pulse).

The number of printed layers and thus the amount of material per area also affects the IPL sintering. In order to sinter all the material from the top surface to the interface with the substrate, the energy has to pass through the printed nanoparticle structure. Figure 8 presents the obtained relative conductivity as a function of layer count. This graph only includes single pulse setups using a bank voltage of 320 V and a pulse duration of 5 ms. Each result represent an average of 12 measurements and 95% confidence intervals are included in the plot.

The most obvious observation from Figure 8 is that patterns printed with one layer enable significantly higher relative conductivities than higher layer counts. No observable difference exists between two and three layers but four layers have lower conductivity, mostly due to cracking. This makes the cracking behavior different than what was observed for laser sintering where one layer patterns had so extensive cracking that the pattern had open circuits. In IPL sintering it is vice versa. This is thought to be because of different energy transfer methods in laser and IPL sintering. In laser sintering small areas are treated at time whereas in IPL sintering comparable large areas are treated at time. This might affect the shrinking and the cracking behavior of the printed patterns with the different sintering methods.

Multilayer patterns have lower relative conductivity but also lower overall resistance due to a larger layer thickness and therefore larger cross-section areas of the conductor line. These results demonstrate, that the IPL sintering strongly depends on the layer thickness. For one layer, the sintering of the copper particles was much better than for two to four layers. Main reason for the behavior is a more intensive crack formation with higher number of layers due to a higher layer thickness.

### Microstructure

**Figure 9:**
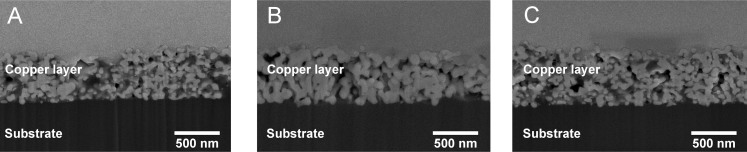
SEM images of the cross-section of IPL-sintered patterns with two layers. The bank voltage and theoretical energy density were kept constant at 320 V and about 8.94 J/cm^2^, respectively. Further details of the test samples are listed in Table 2.

Figure 9 shows cross-sectional SEM images of IPL-sintered test samples at different process parameters. Process parameter details of the test samples are explained in Table 2. All images in Figure 9 are taken from two layer samples and have been processed with a bank voltage of 320 V. All samples are sintered using similar theoretical total energy densities and power settings but the energy was divided in varying number of pulses thus enabling the comparison between single pulse and multi pulse setups. The real energy density varies slightly due to capacitor reload effect explained in Supplementary Information and in this manuscript before.

Cross-sectional SEM images presented in Figure 9 show some minor differences in microstructures of different sintering setups. Figure 9 B) shows more uniformal microstructure with larger grain size. However, the differences in microstructures between Figure 9 A) and B) are significant even though difference in corresponding conductivities is really small. On the other hand, microstructural differences between Figure 9 A) and C) are minimal regardless of a significant difference in respective relative conductivities. This would suggest similar observation as with laser sintering: Cross-sectional SEM images of the microstructure alone cannot be used to deduce the electrical conductivity.

**Figure 10:**
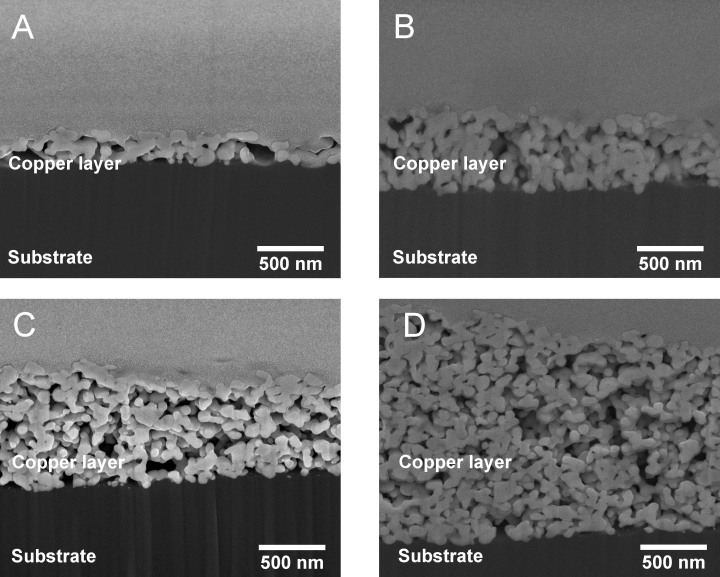
SEM images of the cross-section of IPL-sintered patterns with (A) one, (B) two, (C) three and (D) four layers. All samples are sintered with identical sintering parameters. Further details are listed in Table 3.

The SEM images presented in Figure 10 show clearly the increasing layer thickness as a result of the increasing number of layers from A) about 270 ± 90 nm to B) about 700 ± 80 nm to C) 980 ± 230 nm and finally D) with approximately 1920 ± 260 nm. Details about the sample processing are listed in Table 3. Next to the increasing layer thickness, one can also see decreasing grain sizes from Figure 10 A) to D). The larger grain size in Figure 10 A) enables larger interconnected areas between the copper grains leading to higher relative conductivity. Corresponding relative conductivities are 20.1%, 15.2%, 12.6% and 11.2% of bulk copper conductivity for one, two, three and four layers respectively. The obtained results are similar as presented in Figure 8. Therefore, in this case the electrical conductivity corresponds with the microstructure uniformity.

**Figure 11:**
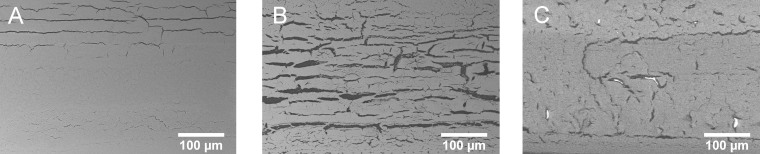
Crack formation in (A) printed and dried (not sintered), (B) IPL sintered and (C) laser sintered samples (four layers).

As already mentioned before, significant cracking was observed in the four layer test samples after IPL sintering. Figure 11 shows a comparison of SEM images taken at the top surface of A) printed and dried, B) IPL sintered and c) laser sintered copper layers (in all cases four layers). Some longitudinal cracking is already present in the not sintered sample at the edges (Figure 11 A), most likely due to solvent evaporation and layer thickness variations. However, it is not as prominent as after IPL or laser treatment. Laser sintering (as seen in Figure 11 C) seems to cause significantly less cracking than IPL sintering (Figure 11 B). We could also see, that the cracking is intensified with increasing number of layers and therefore with increasing layer thickness. Interestingly, the cracking seems to have in this case relatively small effect on the conductivity as a relative conductivity of 12.8% of bulk copper was obtained with the sample in Figure 11 B). This can be explained in terms of the measurement setup. The cracking occurs mainly along printing direction^11^. The printing direction refers in this case also to the measurement direction. Thus, the cracks influence for sure the cross-sectional area, but they do not interrupt the electric current path which would most probably take place when the crack formation changes its orientation.

### Comparison

As the main function of the printed copper ink is to act as an electrical conductor, the overall performance to be focused in this study is the achievable relative conductivity with both sintering techniques.

**Figure 12:**
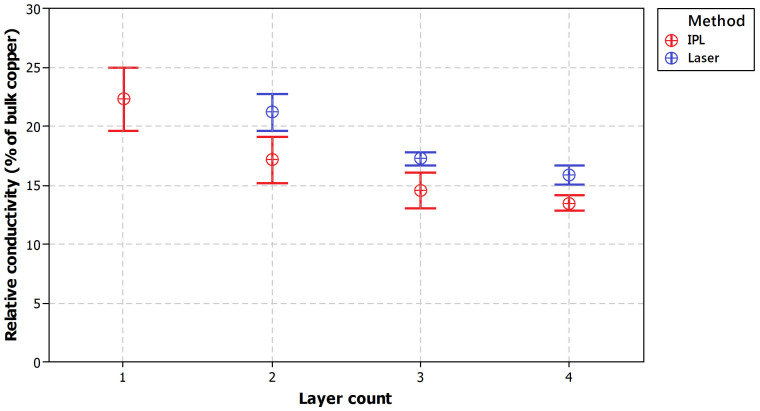
Relative conductivity as a function of layer count for both laser and IPL sintering.

Figure 12 presents the achievable relative conductivities for both laser and IPL sintering technique for different layer counts. Best setup was chosen to represent each sintering method and layer count. The details of the parameters are listed in the Supplementary Information (Table S1 and Table S2). Laser sintering results represent averages of 10 measurement and IPL results averages of 12 measurements and 95% confidence intervals are included in the plot.

Comparable conductivities, over 20% of bulk copper conductivity, can be obtained with both sintering techniques analyzed in this study. Based on the results presented in this contribution, it seems that laser sintering can be used to provide slightly better relative conductivity for each tested layer count, except for single layer patterns which were found to be not processable for laser sintering due to extensive crack formation during the sintering process.

In general, IPL sintering was found to favor thinner layers. Serious cracking issues were observed with four layer samples even though decent conductivities were achieved. With laser sintering, on the other hand, it was not possible to sinter one layer samples. But in all other cases less cracking was observed in comparison to IPL sintering.

### Required energy per area for both techniques

Laser sintering seems to operate with much higher energy densities than IPL sintering. However, one has to take in account that the energy for the IPL sintering is difficult to measure and so the theoretical energy density given by the software was used. We have checked some of the IPL settings with a bolometer and found in all cases slightly lower energies as displayed by the software tool. Nevertheless, based on the values we can see the trend. Laser sintering of the tested copper nanoparticle provided the best results with an energy density of 64.9 J/cm^2^ and the usable range of energy density ranges from 30 J/cm^2^ to 100 J/cm^2^. Meanwhile, the IPL sintering provided the best conductivity results with an energy density of about 6.5 J/cm^2^. The usable energy density range in IPL sintering was about 3 J/cm^2^ to 18 J/cm^2^. Thus, the laser sintering approach required at least about five times higher energy density than IPL sintering but the processing range was much higher.

Inadequate energy density results in low electrical conductivity because no or only weak sintering took place. On the other hand, too much energy results in intensified crack formation and destruction of the printed pattern.

### Microstructure comparison between laser and IPL-sintered samples

Microstructures of test samples sintered with both sintering techniques were also analyzed and major differences were not observed. Laser sintering seems to produce a slightly more uniformal structure than IPL sintering but the difference is insignificant. When comparing microstructures within each sintering technique and analyzing the effect of processing parameters, it was found out that the microstructure does not necessarily correlate with the obtained relative conductivity. Therefore, the microstructure alone cannot be used to estimate the relative conductivity.

## Conclusions

Both of the tested sintering methods were successfully used for the sintering of a copper nanoparticle ink and conductivities of over 20% of bulk copper conductivity were obtained. Therefore, both sintering techniques are in principle suitable for printed electronics manufacturing.

The amount of energy delivered to the printed structure during the sintering process is usually mentioned as the most important factor in sintering publications. However, the results of this study clearly show that the energy is by far not the only dictating factor for the achievable conductivity. More important seems to be, regardless of the used sintering technique, the way the energy is delivered to the pattern to be sintered. Other sintering parameters, such as speed and power of sintering, are also affecting the sintering process and are of high importance, e.g. in roll-to-roll processing where the speed is usually predefined.

It was also noticed, that the determination of electrical conductivity just by the cross-sectional SEM images of the microstructure is not possible in all cases. Correlation of conductivity and microstructure was observed for different printed layer counts but not for different sintering parameters. While the microstructure images are indicative of the level of sintering and of the electrical conductivity, they should not by themselves used as evidence for achieved conductance since e.g. macroscopic phenomena such as crack formation are not considered.

## Experimental Section

### Materials and equipment

The commercially available Intrinsiq CI-002 nanoparticle copper ink with an average particle size of 45 nm and a metal loading of 12 wt% was used in this study. Inkjet printing was carried out on an iTi MDS 2.0 XY deposition system equipped with a Fujifilm Dimatix Spectra SE-128 printhead. The printhead has 128 individually addressable nozzles with a distance of 508 μm arranged in a single line. Each nozzle has a diameter of 30 μm resulting to a nominal drop volume of 30 pL. Before printing, the CI-002 was filtered with a 1.5 μm glass fiber filter, and treated with a Elmasonic S 120 H ultrasonic unit to ensure homogeneous nanoparticle distribution without agglomeration. Finally, the ink was degassed in a low pressure chamber for better jetting stability during printing. Printing was performed with a resolution of 900 dpi which corresponds to a center-to-center distance (drop spacing) of the deposited drops of 28 μm. 3 M Kapton (USA) polyimide films with a thickness of 50 μm were used as substrates due to the good thermal stability which allows testing of high intensity sintering setups.

Sintering of the layers was done using a custom-built laser system and the Novacentrix PulseForge 3200 IPL system. A diode laser module (LIMO HLU-35C10x2-808-CB) with a wavelength of 808 nm and a nominal optical power output of 35 W was applied. The laser beam was adjusted in a rectangular shape of approximately 1.1 × 0.4 mm^2^ and a focal length of 120 mm. Two parameters were varied in this investigation: Laser scanning speed (from 50 to 250 mm/s in steps of 50 mm/s) and laser energy density (from 27.5 to 100 J/cm^2^).

The Novacentrix PulseForge 3200 IPL system integrated in a microFLEX roll-to-roll system (3D-Micromac) was employed for the IPL sintering. It was used in stand-alone operation with the PulseForge 1.1 software tool and allows the emission of adjustable broad-spectrum light pulses. By adjusting the pulse characteristics such the pulse duration (from 30 μs to 10 ms), the number of pulses (1 to 10), its repetition frequency and the lamp driver output voltage (150 to 390 V), the energy density level can be well controlled in a range of about 0.01 J/cm^2^ to more than 15 J/cm^2^.

### Print pattern

**Figure 13:**
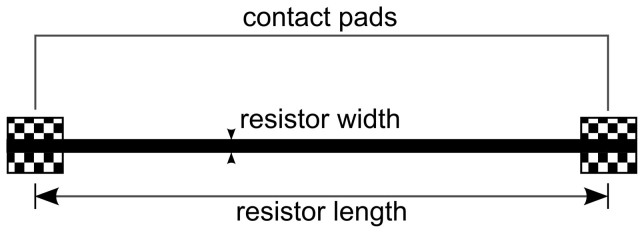
Print pattern used in the experiment. The solid line shows the line resistor printed with one, two, three or four layers. The checkered areas are contact pads for the measurement and were printed with a maximum of two layers.

The experimental tests were done with a simple conductor line as shown in Figure 13. A standard 2-point measurement method was used to determine the resistance. Length of the line where the electrical resistance was measured is 13 mm and the digital line width was 280 μm. When printing the lines, the line width of the pattern is higher than the digital width of 280 μm due to ink spreading. Table 4 summarizes the printing parameters used in this study. The pulses refer in this case to the control signal applied to the piezo-electric inkjet printhead.

To vary the amount of material deposited, (i) one, (ii) two, (iii) three, and (iv) four layers were printed each with a resolution of 900 dpi. In order to prevent excessive flooding during printing of larger areas, the print pattern file was modified so that the checkered areas in Figure 13 are only printed maximum with two layers. When several layers were printed the previously printed layer was allowed with enough drying time on the heated print plate so that the change of color of the ink indicated evaporation of the solvent. This prevents flooding during printing and improves the layer quality and repeatability.

### Characterization

The main focus was set on the electrical performance of the printed and sintered patterns. The resistance of the sintered layers was measured using a two-point measurement system. The measured resistance was compared with the optimum resistance calculated based on the known architecture and amount and known material characteristics of the ink printed. Therefore, the ratio between measured and optimum resistance is called “relative conductivity” and “% of bulk copper” is used as a unit. The relative conductivity is seen as measure of goodness of the sintering process as it tells what portion of the printed copper is taking part in the final conductivity.

SEM imaging was applied to analyze the microstructure of the sintered layers. Focused Ion Beam (FIB) technology was used to cut the printed layers so that cross-sectional images of the layer structure were obtained.

## Author Contributions

J.N. prepared the samples and did the laser sintering. E.S. and H.K. did the IPL sintering. J.N., E.S. and H.K. wrote the main manuscript and the supporting information. M.M. and R.R.B. contributed for results analysis and manuscript writing. All authors reviewed the manuscript.

## Supplementary Material

Supplementary Information Supplementary Information

## Figures and Tables

**Table 1:** Sample details of cross-sectional SEM images of laser-sintered samples

Image	Number of layers	Scanning speed (mm/s)	Energy density (J/cm^2^)	Conductivity (% of bulk copper conductivity)
A	4	50	84.9	15.2
B	4	100	64.9	14.9
C	4	200	51.1	11.8

**Table 2:** Sample details of cross-sectional SEM images of IPL-sintered samples

Image	Number of layers	Bank voltage (V)	Pulse duration (ms)	Number of pulses	Pulse frequency (Hz)	Energy density (J/cm^2^)	Conductivity (% of bulk copper conductivity)
A	2	320	5	1	-	8.94	14.4
B	2	320	2.5	2	10	8.94	15.2
C	2	320	1	5	10	8.94	6.8

**Table 3:** Sample details of cross-sectional SEM images of IPL-sintered samples

Image	Number of layers	Bank voltage (V)	Pulse duration (ms)	Number of pulses	Pulse frequency (Hz)	Energy density (J/cm^2^)	Conductivity (% of bulk copper conductivity)
A	1	320	2.5	2	10	8.94	20.1
B	2	320	2.5	2	10	8.94	15.2
C	3	320	2.5	2	10	8.94	12.6
D	4	320	2.5	2	10	8.94	11.2

**Table 4:** Inkjet printing parameters for the deposition of the copper layers

Parameter	Value
Printhead	Spectra SE-128
Print resolution	900 dpi
Printhead temperature	55°C
Printplate temperature	60°C
Pulse width	8 μs
Pulse rise/fall time	2 μs/2 μs
Ink	Intrinsiq CI-002

## References

[b1] Niittynen J. *et al.* Alternative Sintering Methods Compared to Conventional Thermal Sintering for Inkjet Printed Silver Nanoparticle Ink. Thin Solid Films. 556, 452–459 (2014).

[b2] Perelaer J. *et al.* Plasma and Microwave Flash Sintering of a Tailored Silver Nanoparticle Ink Yielding 60% Bulk Conductivity on Cost-effective Polymer Foils. Adv. Mater. 24, 3993–3998 (2012). 22718319 10.1002/adma.201200899

[b3] Wünscher S., Stumpf S., Perelaer J. & Schubert U. S. Towards single-pass plasma sintering: temperature influence of atmospheric pressure plasma sintering of silver nanoparticle ink. J. Mater. Chem. C. 2, 1642–1649 (2014).

[b4] Kamyshny A., Steinke J. & Magdassi S. Metal-based Inkjet Inks for Printed Electronics. Open Applied Physics Journal. 4, 19–36 (2011).

[b5] Lee D. J. *et al.* Pulsed light sintering characteristics of inkjet-printed nanosilver films on a polymer substrate. J. Micromech. Microeng. 21, 125023 (2011).

[b6] Perelaer J. *et al.* Roll-to-Roll Compatible Sintering of Inkjet Printed Features by Photonic and Microwave Exposure: From Non-Conductive Ink to 40% Bulk Silver Conductivity in Less Than 15 Seconds. Adv. Mater. 24, 2620–2625 (2012). 22488908 10.1002/adma.201104417

[b7] Tobjörk D. *et al.* IR-sintering of ink-jet printed metal-nanoparticles on paper. Thin Solid Films. 520, 2949–2955 (2012).

[b8] Määttänen A. *et al.* Inkjet-Printed Gold Electrodes on Paper: Characterization and Functionalization. Appl. Mater. Interfaces. 4, 955–964 (2012). 10.1021/am201609w 22233965

[b9] Reinhold I. *et al.* Argon plasma sintering of inkjet printed silver tracks on polymer substrates. J. Mater. Chem. 19, 3384–3388 (2009). 10.1002/adma.200901081 21049504

[b10] Hösel M. & Krebs F. C. Large-scale roll-to-roll photonic sintering of flexo printed silver nanoparticle electrodes. J. Mater. Chem. 22, 15683–15688 (2012).

[b11] Kang H., Sowade E. & Baumann R. R. Direct Intense Pulsed Light Sintering of Inkjet-Printed Copper Oxide Layers within Six Milliseconds. ACS Appl. Mater. Interfaces. 6, 1682–1687 (2014). 24433059 10.1021/am404581b

[b12] Halonen E., Heinonen E. & Mäntysalo M. The Effect of Laser Sintering Process Parameters on Cu Nanoparticle Ink in Room Conditions. Optics and Photonics Journal. 3, 35658 (2013).

[b13] Halonen E., Koskinen S., Leino I., Heljo P. & Mäntysalo M. Sintering of Inkjet-printed Cu-nanoparticle Ink in Ambient Conditions Using a Continuous Wave 808 nm Diode Laser. MRS Proceedings. 159, (2013), http://dx.doi.org/10.1557/opl.2013.250.

[b14] Marjanovic N. *et al.* Inkjet printing and low temperature sintering of CuO and CdS as functional electronic layers and Schottky diodes. J. Mater. Chem. 21, 13634–13639 (2011).

