# Supplementary material for: Comparison of laser and intense pulsed light sintering (IPL) for inkjet-printed copper nanoparticle layers
Source: Sci Rep. 2015 Mar 6;5:8832. doi: 10.1038/srep08832 (PMC4351538; doi:10.1038/srep08832)
Supplement: Supplementary Information [file srep08832-s1.doc]

# Supplementary Information -

# Comparison of laser and intense pulsed light sintering (IPL) for inkjet-printed copper nanoparticle layers

Juha Niittynen1, Enrico Sowade2, Hyunkyoo Kang2, Reinhard R. Baumann2,3, Matti Mäntysalo1

1 Department of Electronics and Communications Engineering, Tampere University of Technology, Tampere, Finland.

2 Digital Printing and Imaging Technology, Technische Universität Chemnitz, Chemnitz, Germany

3 Department Printed Functionalities, Fraunhofer Research Institute for Electronic Nano Systems (ENAS), Chemnitz, Germany

**IPL sintering: Influence of pulse frequency on energy density**

Applying multi pulses with high frequencies will result in decreasing energy density from pulse to pulse due to the short time delay between the single pulses. In contrast, with a lower frequency higher energy densities can be obtained. The reasons are mainly the electrical mechanism of the IPL system. The flash is generated by accumulation of high electric charges in a capacitor. Charging and discharging requires time and thus for higher frequencies the charging with the adjusted and targeted voltage is not possible – instead a slightly lower voltage will be applied resulting is lower energy density.

However, when applying high frequencies the temperature within the sample can rise up with each single flash even so the energy density decreases from flash to flash. For lower frequencies the sample will heat and cool down with each flash and thus the temperature within the sample is similar for each flash an in total lower than for higher frequencies.

Figures S1 and S2 depict schematically the explained dependency of frequency on sample temperature and irradiance. The energy density is determined by flash length integration of the irradiance. Thus, the energy density corresponds to the area under the blue line. Figure S1 shows an example of pulses with high frequency. The temperature within the sample increases with each pulse. On the other hand, the irradiance energy and thus the energy decreases with each pulse. Figure S2 shows an example of pulses with low frequency. The temperature within the sample remains constant due to a long cooling period between the pulses (low frequency). Also, the irradiance and thus the energy density remains constant due to sufficient time for the charging and discharging of the capacitor of the IPL system. The maximum temperature within the sample is in the case of Figure S2 obviously lower than in the case of Figure S1.


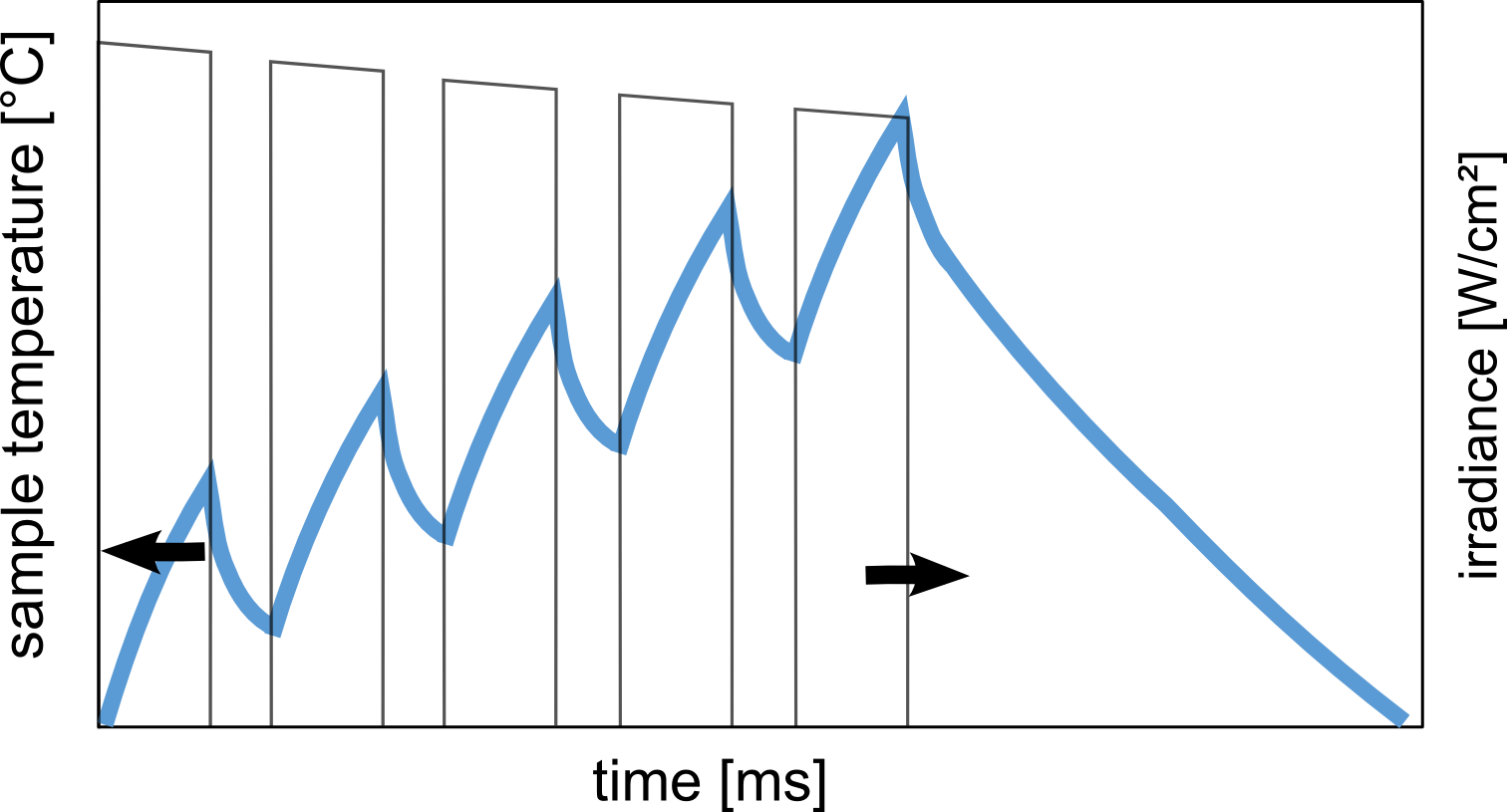


**Figure S1: Temperature of the sample and irradiance energy as a function of time for high frequency pulses**


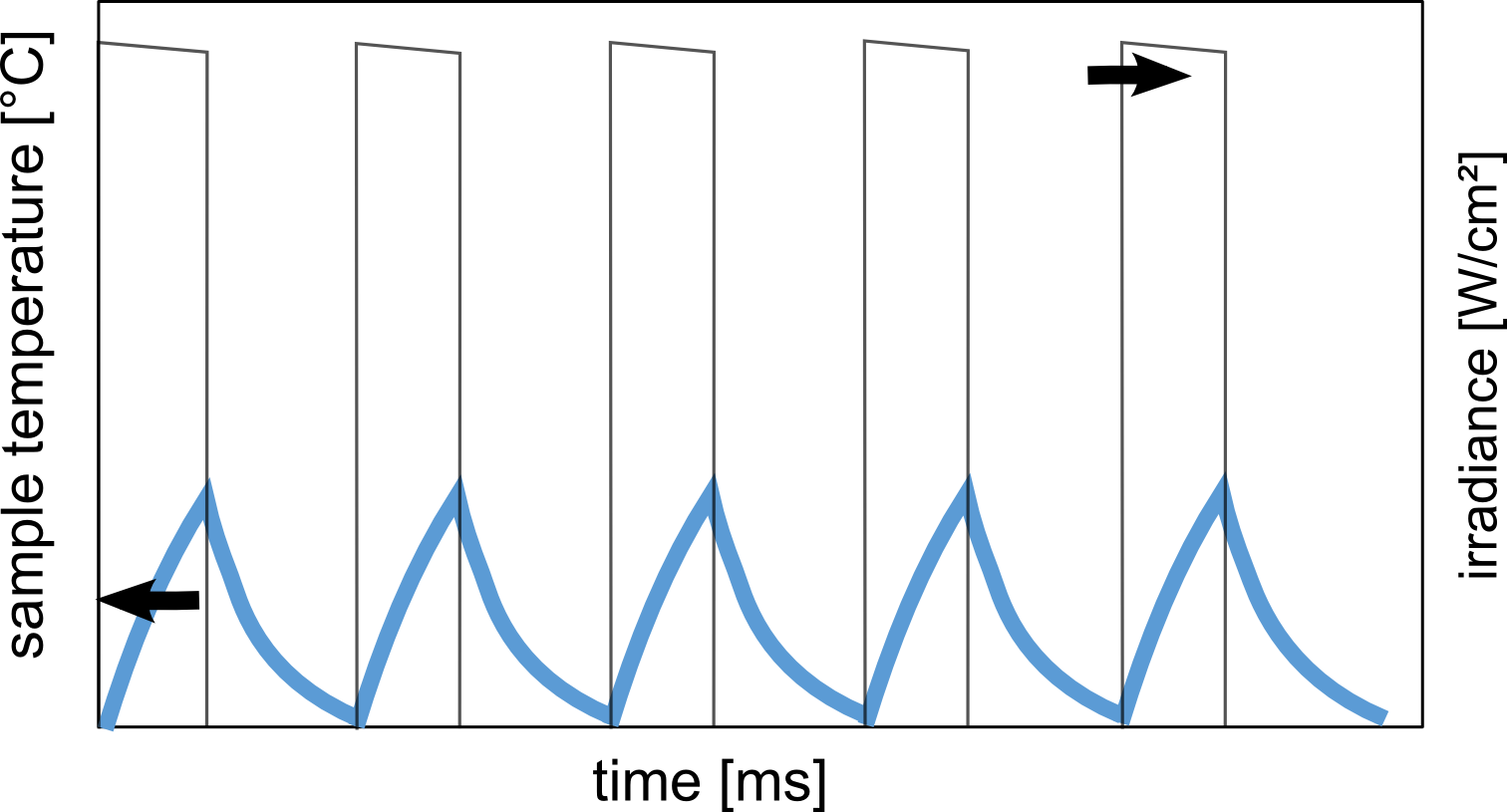


**Figure S2: Temperature of the sample and irradiance energy as a function of time for low frequency pulses**

**IPL sintering: Influence of number of pulses on energy density**

Figures S3 and S4 show schematic graphs of the influence of the number of pulses with the same total duration on the irradiance. In Figure S3 one single pulse is applied with a total duration of 5 ms. In Figure S4 two pulses are applied each with 2.5 ms duration resulting in the same overall pulse duration of 5 ms for both settings. The energy density is determined by the flash length integration of the irradiance. Thus, the energy density corresponds to the area under the blue line. Due to the slope of the pulse with time, the energy density in Figure S4 (two pulses with 2.5 ms) is slightly higher than the energy density in Figure S3 (single pulse with 5 ms).


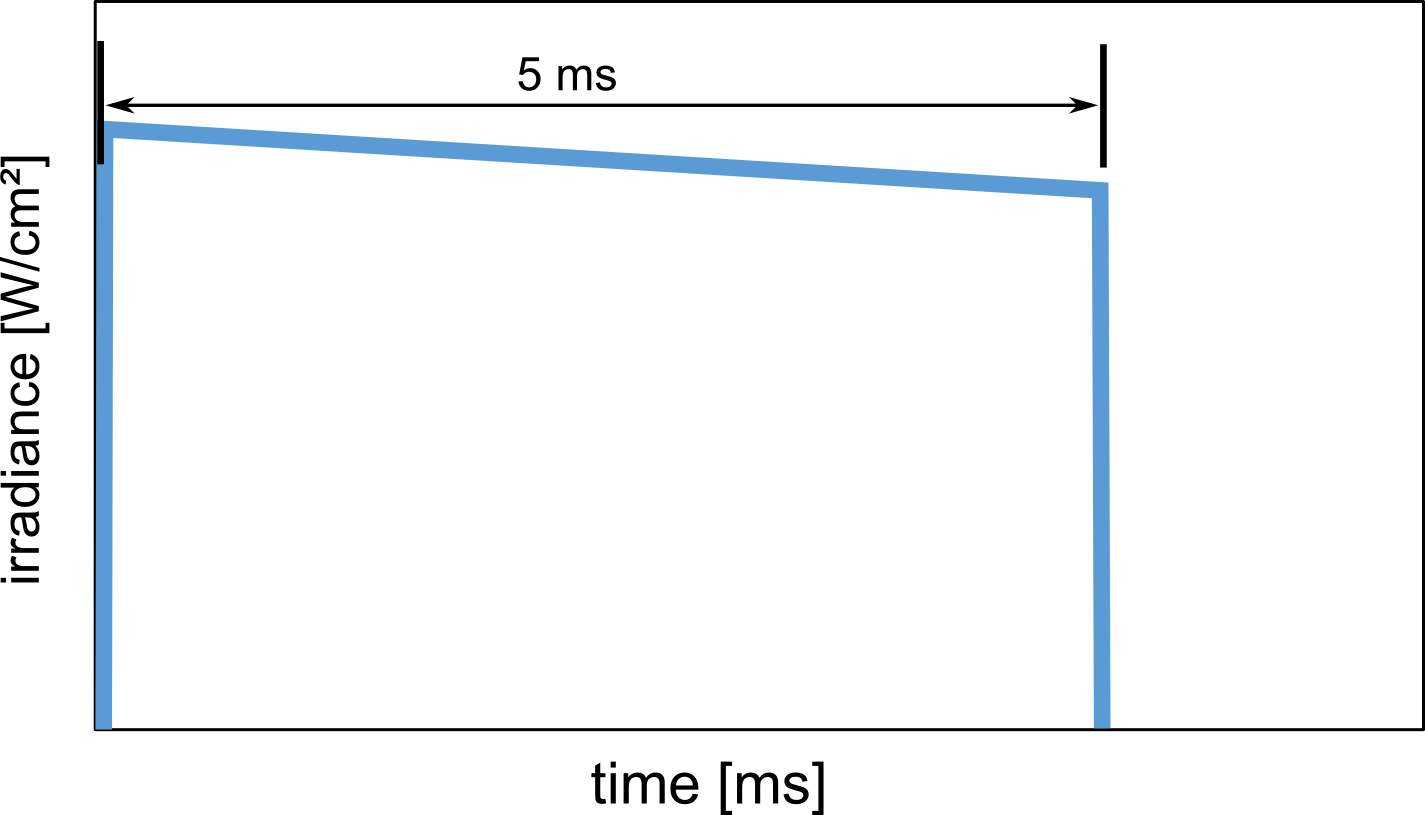


**Figure S3: Irradiance as a function of time for a single pulse of 5 ms**


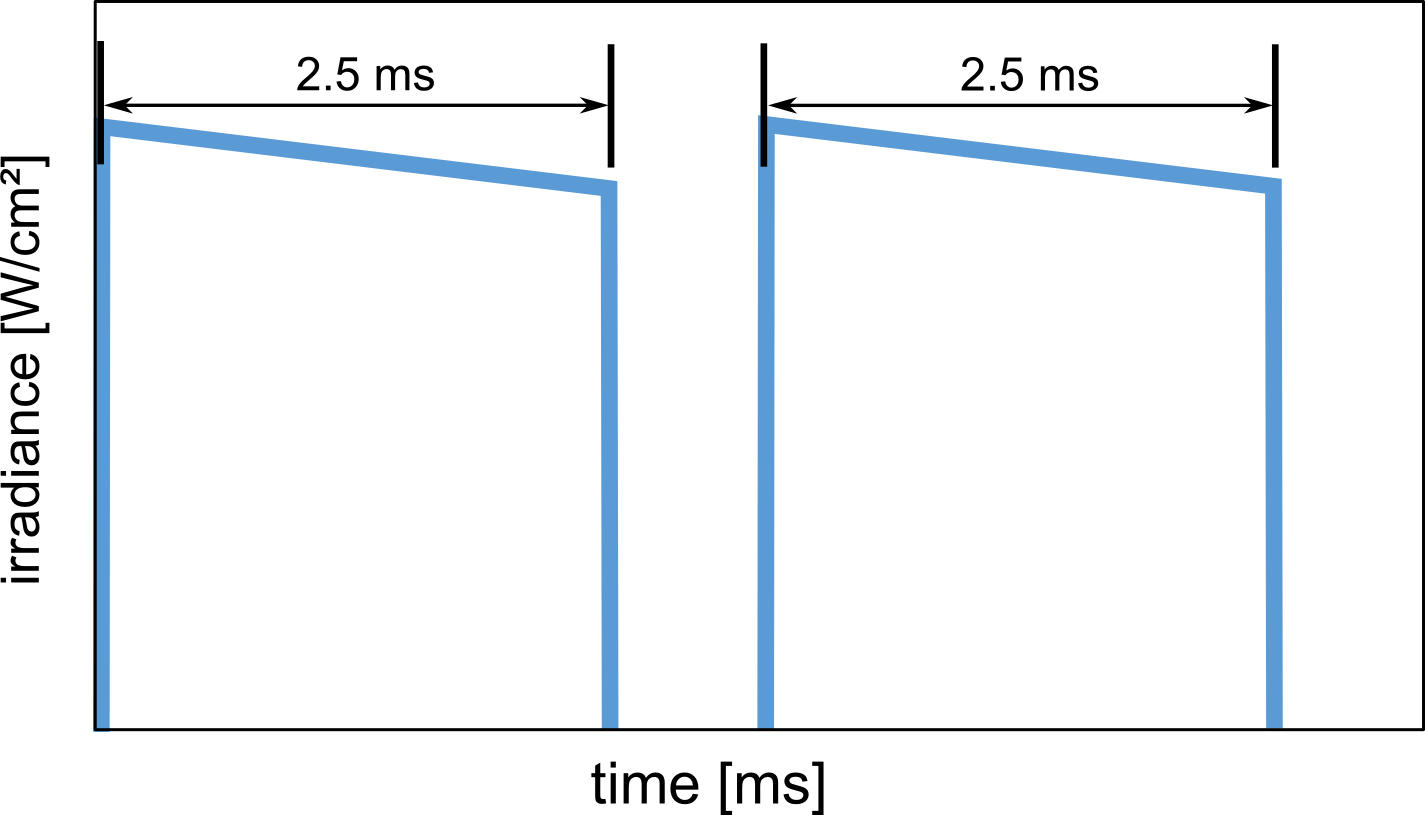


**Figure S4: Irradiance as a function of time for two pulses each with 2.5 ms and thus total again 5 ms**

**Laser sintering and IPL sintering: Detailed parameter information for values of Figure 12.**

Table S1: Laser sintering parameters for values of Figure 12.

| Number of layers | Optical power (W) | Energy density (J/cm^2^) |
| --- | --- | --- |
| 2 | 13.12 | 57.4 |
| 3 | 14.83 | 64.9 |
| 4 | 13.12 | 57.4 |

Table S2: IPL sintering parameters for values of Figure 12.

| Number of layers | Bank voltage (V) | Pulse duration (ms) | Number of pulses | Energy density (J/cm^2^) |
| --- | --- | --- | --- | --- |
| 1 | 300 | 8 | 1 | 6.5 |
| 2 | 325 | 6 | 1 | 6.9 |
| 3 | 320 | 5 | 1 | 6.0 |
| 4 | 320 | 6 | 1 | 6.6 |
